# 108 Why do we run? Motivation profiles and training characterstics in the Garmin-RUNSAFE Running Health Study

**DOI:** 10.1093/eurpub/ckae114.194

**Published:** 2024-09-26

**Authors:** Sebastian Skejø, Chloe T Blacket, Rasmus Østergaard Nielsen, Joel T Fuller, Hunter Bennett, Alyson J Crozier, John B Arnold

**Affiliations:** Research Unit for General Practice, Aarhus, Denmark; Department of Public Health, Aarhus University, Aarhus, Denmark; Alliance for Research in Exercise, Nutrition and Activity (ARENA), Allied Health & Human Performance, University of South Australia, Adelaide, Australia; Research Unit for General Practice, Aarhus, Denmark; Department of Public Health, Aarhus University, Aarhus, Denmark; Department of Health Sciences, Faculty of Medicine, Health and Human Sciences, Macquarie University, Sydney, Australia; Alliance for Research in Exercise, Nutrition and Activity (ARENA), Allied Health & Human Performance, University of South Australia, Adelaide, Australia; Alliance for Research in Exercise, Nutrition and Activity (ARENA), Allied Health & Human Performance, University of South Australia, Adelaide, Australia; Alliance for Research in Exercise, Nutrition and Activity (ARENA), Allied Health & Human Performance, University of South Australia, Adelaide, Australia

## Abstract

**Purpose:**

To identify differences in training between runners with different motivation profiles.

**Methods:**

We invited runners enrolled in the Garmin-RUNSAFE Running Health Study to answer the Behavioural Regulations in Exercise Questionnaire, version 3 (BREQ-3) – a 24-item, six subscale questionnaire assessing motivation.

For each runner, we computed the mean weekly number of running sessions (Mfreq), the mean weekly running distance (Mkmwk), the mean weekly running duration (Mdurwk) and the average pace (Mminkm) in the four weeks preceding BREQ-3 completion.

We identified motivation profiles using latent profile analysis on the BREQ-3 subscale scores and compared profiles’ training using Kruskal-Wallis and Dunn’s test with Bonferroni correction with statistical significance set at p < 0.05.

**Results:**

2278 runners completed the BREQ-3. We identified four motivation profiles: ‘balanced engagers’ (n = 620, Mfreq = 2.6, Mkmwk = 23.6 km, Mdurwk = 2.3 hours, Mminkm = 6.15 min/km) with moderate-to-high levels of autonomous motivation; ‘autonomy achievers’ (n = 1379, Mfreq = 3.4, Mkmwk = 31.3 km, Mdurwk = 3.1 hours, Mminkm = 6.10 min/km) with high levels of autonomous motivation; ‘reluctant runners’ (n = 117, Mfreq = 1.9, Mkmwk = 13.2 km, Mdurwk = 1.4 hours, Mminkm = 6.46 min/km) with low levels of autonomous motivation; and ‘controlled engagers’ (n = 162, Mfreq = 2.9, Mkmwk = 24.5 km, Mdurwk = 2.5 hours, Mminkm = 6.38 min/km) with high amotivation and extrisinc motivation, but also high autonomous motivation.

‘Autonomy achievers’ had higher Mfreq, Mkmwk, Mdurwk and Mminkm compared to all other profiles (p < 0.001), while ‘reluctant runners’ had lower Mfreq, Mkmwk and Mdurwk than all other profiles (p < 0.001).

**Conclusions:**

Running is one of the most popular forms of physical activity. Our findings show that runners have different motivation profiles which are associated with distinct training patterns. Therefore, it may be important to consider runners’ motivational orientation when designing policies and interventions aimed at enhancing physical activity through running.

**Support:**

The study is supported by unconditional grants from Aarhus University Research Foundation (grant number: AUFF-E-2015-FLS-9-9) and the Danish Rheumatism Association (grant number: R160-A5157).

